# A Battery SOC Estimation Method Based on AFFRLS-EKF

**DOI:** 10.3390/s21175698

**Published:** 2021-08-24

**Authors:** Ming Li, Yingjie Zhang, Zuolei Hu, Ying Zhang, Jing Zhang

**Affiliations:** 1 College of Information Science and Engineering, Hunan University, Changsha 410000, China; huzuolei@hnu.edu.cn; 2 School of Computer Science, Northwestern Polytechnical University, Xi’an 710000, China; ying_zhang@nwpu.edu.cn; 3 Institute of Industry Energy-Saving Control and Evaluation, Hunan University, Changsha 410000, China; zhangj@hnu.edu.cn

**Keywords:** battery state of charge, parameter estimation, recursive least square, extended Kalman filtering

## Abstract

The lithium-ion battery is the key power source of a hybrid vehicle. Accurate real-time state of charge (SOC) acquisition is the basis of the safe operation of vehicles. In actual conditions, the lithium-ion battery is a complex dynamic system, and it is tough to model it accurately, which leads to the estimation deviation of the battery SOC. Recursive least squares (RLS) algorithm with fixed forgetting factor is widely used in parameter identification, but it lacks sufficient robustness and accuracy when battery charge and discharge conditions change suddenly. In this paper, we proposed an adaptive forgetting factor regression least-squares–extended Kalman filter (AFFRLS–EKF) SOC estimation strategy by designing the forgetting factor of least squares algorithm to improve the accuracy of SOC estimation under the change of battery charge and discharge conditions. The simulation results show that the SOC estimation strategy of the AFFRLS–EKF based on accurate modeling can effectively improve the estimation accuracy of SOC.

## 1. Introduction

The main purpose of the battery management system (BMS) is to ensure the safe operation of the batteries [1]. State evaluation of a battery, including state of charge, state of health, and state of life, is a critical task for a BMS. As the power source and energy storage equipment in the actual operation of electric vehicles, the battery will carry out continuous charging and discharging operations, and the battery state remains changing. It is very important to predict the battery status in advance to adjust the battery management system in time and ensure the safe operation of the battery. As a key power source for hybrid electric vehicles, accurate acquisition of SOC is very important to improve the dynamic performance of the battery and optimize the energy management strategy [2,3]. Model-based SOC estimation is an important research direction. However, battery SOC is affected by many factors such as actual working conditions, ambient temperature, battery aging, and self-discharge rate [4], accurate modeling is difficult, which leads to the difficult problem of high-precision SOC estimation.

There is much research literature on battery SOC estimation methods. Ampere-hour measurement method is one of the traditional SOC estimation algorithms. Due to the unknown initial value of SOC and the existence of an integral part, the estimation error will gradually accumulate with the increase of the battery running time, so the ampere-hour measurement method is usually combined with other estimation algorithms [5,6]. The open-circuit voltage method [7] and the internal resistance characteristic method [8] have a good performance, however, it requires the battery to stand for a period, which is not conducive to online calculation. The estimation accuracy of battery SOC is also affected by actual battery working conditions. The extended Kalman filter (EKF) has been successfully applied for the estimation of SOC in HEV BMSs. The traditional Kalman filter (KF) is used for linear problems, while EKF linearizes the prediction by using partial derivatives and Taylor series expansion [9]. The influence of acquisition accuracy of voltage, current, and other signals on SOC estimation is discussed in the view of the hardware [10]. To reduce the initial error of the Coulomb counting method (CCM), the SOC can be calculated accurately by applying the battery efficiency to the open-circuit voltage (OCV) [11]. The application of machine learning (ML) in the BMS of LIB has long been adopted for efficient, reliable, accurate prediction of several important states of LIB such as state of charge, state of health, and remaining useful life [12]. Electromagnetic interference (EMI) of battery management systems (BMSs) will cause measurement errors of current and voltage signals, which will affect the performance of BSM [13,14,15,16,17,18,19]. For the situation that battery working conditions change rapidly, a recursive calculation method based on the Kalman filter is adopted [20,21,22]. The algorithm considers the battery as a dynamic system, and the filter performs state recursion according to the input (current, voltage, temperature, etc.) to obtain the estimated value of SOC. The method has a strong suppression effect on noise and is suitable for the condition of rapid current variation. However, the algorithm involves complex matrix inversion operation, and the calculation accuracy depends on the precise battery model. As the temperature changes and the battery ages, the internal and external characteristics of the battery will change. To improve the accuracy of the battery dynamic model, it is necessary to identify the battery parameters online [23]. The recursive least squares (RLS) algorithm is an easy algorithm to implement. However, with the increase of data, phenomena such as data saturation will occur, and it cannot be used for parameter identification well. Moreover, due to its fixed forgetting factor, the robustness of the system is poor when disturbed [24]. The least-square algorithm with a forgetting factor (FFRLS) adds a forgetting factor on the basis of RLS algorithm to solve the problem of data saturation [25,26]. Battery parameter identification based on RLS and SOC estimation algorithm based on EKF is widely used [27,28,29,30]. In Literature [27], the model parameter identification deviation caused by current and voltage measurement noise was compensated to improve the parameter identification accuracy to improve the SOC estimation accuracy. In literature [28], decoupled double estimators were used to estimate SOC and battery capacity, and different time scales were used to improve the accuracy and stability of the model. A SOC estimation algorithm combining variable factor RLS and CKF was proposed in the literature [29] to effectively improve the accuracy of estimation. Considering SOC constraints and estimation errors, the literature [30] introduced gain factors based on EKF algorithm, and the proposed algorithm has good effects in terms of accuracy, convergence speed, and robustness.

However, none of the above methods focuses on the SOC estimation under the condition of battery charging and discharging state changes. When the current charging and discharging state changes, the battery system is disturbed, resulting in inaccurate parameter estimation which affects the accuracy of the modeling of the equivalent circuit of the battery, thus affecting the accuracy of battery SOC estimation. In order to improve the estimation accuracy of battery SOC, a precise modeling method based on parameter identification was proposed under the condition of battery charging and discharging state changes. An adaptive forgetting factor regression least-squares algorithm (AFF–RLS) was designed to improve the model parameter identification accuracy, and then the battery SOC is estimated by the extended Kalman filter algorithm.

The main contributions of this paper are as follows:

1. The paper proposed an AFFRLS–EKF SOC estimation strategy based on parameter identification modeling aiming at the uncertainty of battery model parameters under the condition of abrupt change of battery charge and discharge.
2. The second-order Thevenin equivalent circuit model (2-order ECM) of the battery was established, and the SOC–OCV relationship was obtained. According to the charging and discharging conditions, a segment-adaptive recursive least square algorithm was designed to identify the parameters to improve the model accuracy.
3. The proposed estimation strategy is applied to numerical simulation experiments, and RLS–EKF and AFFRLS–EKF are compared. The latter one has better performance in accuracy and robustness.

## 2. Equivalent Circuit Model

**Figure 1:**
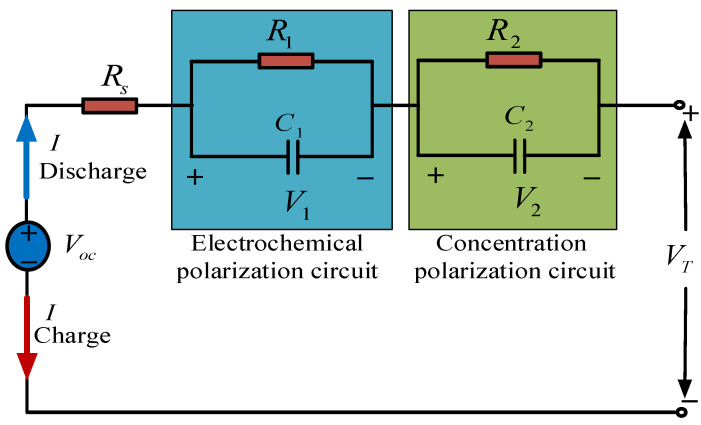
Thevenin equivalent circuit model.

The battery is a complex electrochemical system. An accurate description of battery internal and external characteristics is one of the solutions to improve the accuracy of battery SOC estimation. In order to accurately describe the battery characteristics, the electrochemical model [31,32,33], equivalent circuit model [34,35,36], electrochemical impedance model [10], and other model-based methods were proposed to solve the SOC estimation problem. The more accurate the battery model is to simulate the electrochemical processes that occur during the battery operation, the more accurate the model will be. The equivalent circuit model has been widely used due to its better model accuracy and higher computational efficiency [37]. Combined with the model accuracy, calculation amount, and feasibility analysis, the equivalent circuit model second-order Thevenin model is adopted. The second-order RC model can simulate the electrochemical polarization and concentration polarization of the battery, which is one of the most common models of lithium batteries in the actual operation of vehicles. Compared with the first-order Thevenin model, it can more accurately describe the actual characteristics of the battery. Figure 1 shows a second-order Thevenin equivalent circuit model.

As shown in Figure 1, when current I flows out of the positive electrode, the battery is in a state of discharge; otherwise, the battery is in a state of charge. $V_{oc}$ is the battery’s open-circuit voltage (OCV) which represents the nonlinear relation with the SOC of the battery. $R_{s}$ is the ohmic resistance of the battery that represents the contact resistance between the electrode material and the electrolyte. The first RC network describes the polarization characteristics with $R_{1}$, $C_{1}$ standing for polarization resistance and polarization capacitance, respectively. The second RC circuit illustrates the dynamic behavior of the battery along with concentration polarization, in which $R_{2}$, $C_{2}$ represents the concentration polarization resistance and capacitance, respectively. $V_{T}$ is the terminal voltage that can be measured directly.

We can fit the OCV–SOC relationship by using the OCV–SOC test, and the test results are shown in Table 1 [38].

Literature [39] studied the influence degree and computational complexity of temperature, data points, and aging degree on the lithium battery open-loop voltage model. The 11th-order polynomial fitting was selected, and the functional relationship between VOC and SOC was obtained as follows:

(1) $$\begin{array}{ll} V_{oc}(SOC)= & [0.3890SOC^{11}-2.0452SOC^{10}+4.6445SOC^{9}-5.9667SOC^{8}\\ & +4.7723SOC^{7}-{2.4677SOC}^{6}+0.8329SOC^{5}-0.1832SOC^{4}\\ & +0.0266SOC^{3}-0.0027SOC^{2}+{0.0002SOC}^{1}+0.0003SOC^{0}]\times 10^{5} \end{array}$$

**Figure 2:**
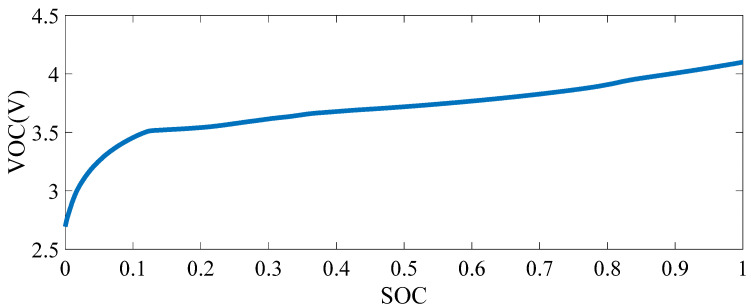
Fitted Map of VOC–SOC.

The curve-fitted SOC–OCV correlation are shown in Figure 2.

According to Kirchhoff’s voltage law, the circuit terminal voltage $V_{T}$ is expressed as follows:

(2) $$V_{T}=V_{OC}-R_{s}I-V_{1}-V_{2}$$

where $V_{1}$ represents the battery electrochemical polarization voltage, which is described as Equation (3). $V_{2}$ represents the battery concentration polarization voltage, which is described as Equation (4).

(3) $$\frac{dV_{1}}{dt}=(\frac{1}{C_{1}})I-(\frac{1}{R_{1}C_{1}})V_{1}$$

(4) $$\frac{dV_{2}}{dt}=(\frac{1}{C_{2}})I-(\frac{1}{R_{2}C_{2}})V_{2}$$

After modeling the Li-battery with Equations (2)–(4), the Li-battery model can be expressed in state-space equation form as follows:

(5) $$\{\begin{array}{l} \left[\begin{array}{l} \frac{dSOC}{dt}\\ \frac{dV_{1}}{dt}\\ \frac{dV_{2}}{dt} \end{array}\right]=[\begin{array}{ccc} 0 & 0 & 0\\ 0 & \left(1+\frac{-1}{R_{1}C_{1}}\right) & 0\\ 0 & 0 & \left(1+\frac{-1}{R_{2}C_{2}}\right) \end{array}]+\left[\begin{array}{c} \left(\frac{-1}{C}\right)\\ \left(\frac{1}{C_{1}}\right)\\ \left(\frac{1}{C_{2}}\right) \end{array}\right]I\\ V_{T}=V_{OC}-R_{s}I-V_{1}-V_{2} \end{array}$$

where *C* represents the capacity of the Li-battery (Ah).

The battery model parameters are greatly affected by the system model and external disturbance. The RLS algorithm can accurately capture the real-time characteristics of the system by regularly correcting and updating the system parameters. Therefore, Equation (5) can be expressed in the s-domain as follows:

(6) $$G(s)=\frac{V_{T}(s)-V_{oc}(s)}{I(s)}=-\left[R_{s}+\frac{R_{1}}{1+R_{1}C_{1}s}+\frac{R_{2}}{1+R_{2}C_{2}s}\right]$$

where *s* is for the frequency operator.

To ensure the consistency of system stability before and after transformation, the function was transformed from the *s* domain to the *z* domain by the bilinear transformation method.

(7) $$s=\frac{2}{T}\frac{1-z^{-1}}{1+z^{-1}}$$

Substitute Equation (7) into Equation (6) and simplify to obtain the discrete transfer function of the system. Matlab solves the change from the s domain to the *z* domain to obtain the transfer function of *Z* domain, as shown in Equation (8).

(8) $$G(z)=\frac{b_{0}+b_{1}z^{-1}+b_{2}z^{-2}}{1-a_{1}z^{-1}-a_{2}z^{-2}}$$

where $\left\{b_{i}|i=0,1,2\right\}\;$, $\left\{a_{j}|j=1,2\right\}\;$ are the parameters to be identified. The relationship between model parameters and parameters to be identified is as follows:

(9) $$\left\{ \begin{array}{l} R_{s}=-\frac{b_{0}-b_{1}+b_{2}}{a_{1}-a_{2}+1}\\ R_{s}+R_{1}+R_{2}=\frac{b_{0}+b_{1}+b_{2}}{a_{1}+a_{2}-1}\\ R_{1}C_{1}R_{2}C_{2}=-\frac{T^{2}(a_{1}-a_{2}+1)}{4(a_{1}+a_{2}-1)}\\ R_{1}C_{1}+R_{2}C_{2}=-\frac{T(a_{2}+1)}{a_{1}+a_{2}-1}\\ R_{1}C_{1}(R_{s}+R_{2})+R_{2}C_{2}(R_{s}+R_{1})=-\frac{T(b_{0}-b_{2})}{a_{1}+a_{2}-1} \end{array}\right.$$

*T* is the sample time.

Equation (8) can be rewritten in the difference equation form as:

(10) $$E(k)=a_{1}E(k-1)+a_{2}E(k-2)+b_{0}I(k)+b_{1}I(k-1)+b_{2}I(k-2)+\varepsilon (k)$$

where $E(k)=V_{T}(k)-V_{oc}(k)$.

Therefore, Equation (10) can be rewritten as follows in matrix form:

(11) $$y(k)=\theta {\left(k\right)}^{T}\phi (k)+\varepsilon (k)$$

with,

(12) $$\theta (k)={\left[a_{1}\;a_{2}\;b_{0}\;b_{1}\;b_{2}\right]}^{T}$$

(13) $$\phi (k)={\left[E(k-1)\;E(k-2)\;I(k)\;I(k-1)\;I(k-2)\right]}^{T}$$

Equations (10)–(13) will be used in the RLS algorithm to estimate model parameters. Then the battery model parameters can be obtained by Equation (9) after θ(k) is estimated.

## 3. Adaptive Forgetting Factor Regression Least Squares

RLS can reduce the influence of application environment uncertainty on the system model and model parameters by periodic parameter correction and updating, to achieve the accurate acquisition of real-time characteristics of the system. In the RLS algorithm, the forgetting factor is a very important parameter, whose value will affect the convergence rate and sensitivity to noise of the algorithm [24]. Therefore, in this paper, the forgetting factor adaptive recursive least square method is used to estimate the parameters. When the battery charge and discharge conditions change suddenly, the adaptive forgetting factor is introduced to adjust the confidence ratio of the recursive model to the old data and the new data, to realize the accurate estimation of the model parameters. For the system model to be identified as shown in Equation (11), the recursion formula of the recursive least squares algorithm with forgetting factor is described as Equation (14) [40]:

(14) $$\left\{ \begin{array}{l} \hat{\theta }(k+1)=\hat{\theta }(k)+K(k+1)\left[y(k+1)-\varphi^{T}(k+1)\hat{\theta }(k)\right]\\ K(k+1)=\frac{P(k)\varphi (k+1)}{\lambda +\varphi^{T}(k+1)P(k)\varphi (k+1)}\\ P(k+1)=\frac{1}{\lambda }\left[I-K(k+1)\varphi^{T}(k+1)\right]P(k) \end{array}\right.$$

where, *k* = 1, 2, 3, …, $\hat{\theta }(k)$ and $\hat{\theta }(k+1)$ are the identifiable vectors of order k and order *k* + 1 respectively, I is the identity matrix, λ is a constant forgetting factor in the range of 0 to 1, K(k+1) is the correction gain vector, P(k+1) and P(k) are the covariance matrices of order k and order *k* + 1, respectively.

When the battery charging and discharging conditions change suddenly, the system disturbance is large, and the instantaneous error of the parameters to be estimated is large, which affects the stability of the system. Therefore, the forgetting factor λ is designed to reduce the instantaneous error of the estimated parameters and increase the stability of the system. Since λ varies within the range of 0–1, the higher its value is, the stronger the anti-interference ability of the system will be. When the current change exceeds a certain value, it indicates that the current changes from charging state to discharging state or from discharging state to charging state. The rules for detecting current zero crossing are as follows:

(15) $$\left\{ \begin{array}{ll} \delta =0, & \;\;\;\frac{1}{2}\left|I(k)-I(k-1)\right|>\min(\left|I(k)\right|,\left|I(k-1)\right|)\\ \delta =1, & \;\;\;\frac{1}{2}\left|I(k)-I(k-1)\right|\leq \min(\left|I(k)\right|,\left|I(k-1)\right|) \end{array}\right.$$

where, δ indicates whether the charging state of the battery has changed, δ=1 represents the battery changing from charging state to discharging state or the battery changing from discharging state to charging state, δ=0 indicates that the battery remains charged or always remains discharged, min(|I(k)|,|I(k−1)|) is the smaller of |I(k)| and |I(k−1)|. In order to increase the robustness of the system, an adaptive weighting factor λ(k) is introduced, which is adjusted according to the status of the current charge and discharge and the magnitude of identification error

(16) $$\lambda (k)=\left\{ \begin{array}{ll} \;1-\frac{e^{2}(k-1)}{1+\varphi^{T}(k-1)P(k-1)\varphi (k-1)}, & \delta =0\\ \;\lambda =1, & \delta =1 \end{array}\right.$$

where $e(k-1)=y(k-1)-\varphi^{T}\left(k-1\right)\hat{\theta }(k-1)$.

Extended Kalman filter (EKF) can make the optimal estimation of the target state under the minimum variance, which is often used in SOC estimation of lithium iron phosphate batteries. For the nonlinear system, the state equation of discrete space is as follows:

(17) $$\left\{ \begin{array}{l} x_{k}=f(x_{k-1},u_{k-1})+\omega_{k-1}\\ z_{k}=h(x_{k})+\upsilon_{k} \end{array}\right.$$

where $x_{k}$ represents the state of the system at time *k*, $x_{k}\in R^{n}$, $u_{k}$ represents the control variable, $z_{k}$ represents the measurement vector, $z_{k}\in R^{n}$, $\omega_{k}$ and $\upsilon_{k}$ represent process noise and measurement noise, respectively, $\omega_{k}$ and $\upsilon_{k}$ are uncorrelated and subject to Gaussian distribution, $Q_{k}$ and $R_{k}$ are covariance, f(·) and h(·) are nonlinear functions.

Firstly, the nonlinear functions f(·) and h(·) are linearized,

(18) $$A_{k}=\frac{\partial f}{\partial x_{k}}=\left[\begin{array}{ccc} 1 & 0 & 0\\ 0 & e^{-\frac{T}{R_{1}C_{1}}} & 0\\ 0 & 0 & e^{-\frac{T}{R_{2}C_{2}}} \end{array}\right]$$

(19) $$C_{k}=\frac{\partial g}{\partial x_{k}}=\left[\frac{\partial V_{T}}{\partial z}{|}_{z=z_{k}}\;\;\;\;\;\;\;\;\;\;-1\;\;\;\;\;\;\;\;\;-1\right]$$

The extended Kalman filter algorithm is shown in Table 2.

**Figure 3:**
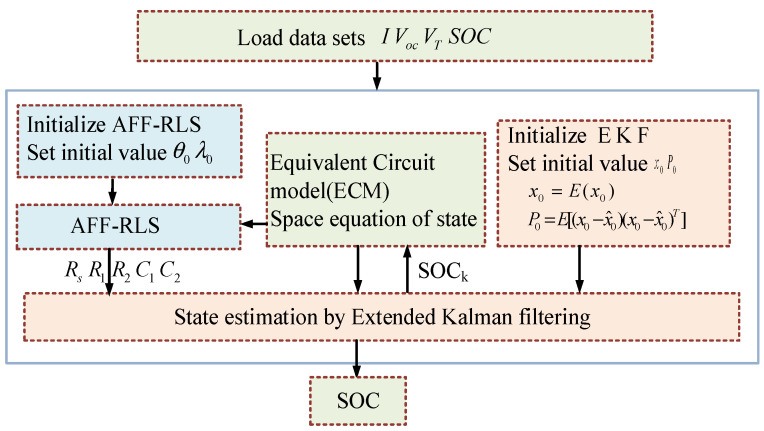
Adaptive forgetting factor regression least-quares–extended Kalman filter (AFFRLS–EKF) algorithm flow chart.

In combination with the forgetting factor adaptive least squares parameter identification and EKF algorithm, the battery SOC estimation strategy is shown in Figure 3.

## 4. Simulation Results and Analysis

**Figure 4:**
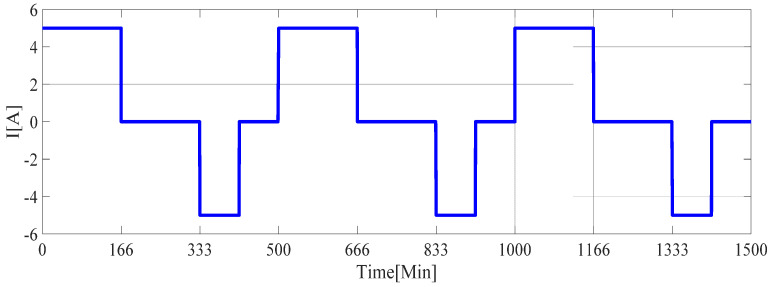
Current curve.

**Figure 5:**
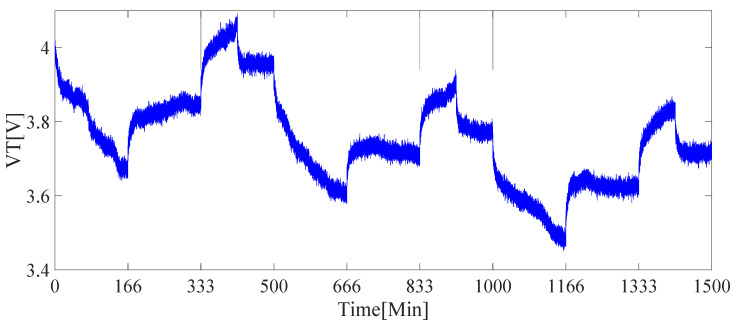
Terminal voltage curve.

The current curve is shown in Figure 4, and the terminal voltage curve is shown in Figure 5. The current remains positive during 0 to 166 min, which indicates the battery is in the discharge state. When *t_i_* = 166 min, the state of the battery changes from the discharge state changes to the open-circuit state. When *t_i_* = 333 min, the battery changes from open-circuit state to charging state. The battery state changes at *t_i_* = 416 min, 500 min, 666 min, 833 min, 916 min, 1000 min, 1166 min, 1333 min, and 1416 min, respectively. The battery terminal voltage curve is shown in Figure 4, In the battery charging time interval, the measured value of the battery terminal voltage increases, while the voltage at the end of the discharge interval decreases. In the open circuit interval of the battery, the terminal voltage remains unchanged, with slight changes due to the soft changes in the terminal voltage caused by the discharge phenomenon in the *R_1_C_1_* circuit and the *R_2_C_2_* circuit.

**Figure 6:**
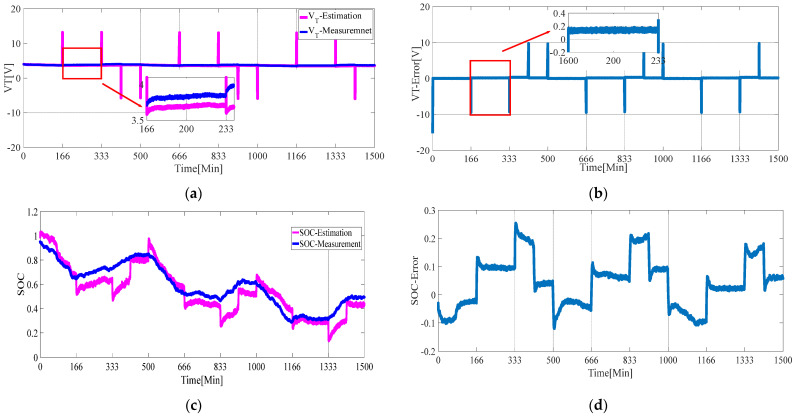
Regression least-squares–extended Kalman filter (RLS–EKF) algorithm. (**a**) Estimated terminal voltage and measured terminal voltage; (**b**) estimated terminal voltage error; (**c**) estimated state of charge (SOC)and measured SOC; (**d**) estimated SOC error.

**Figure 7:**
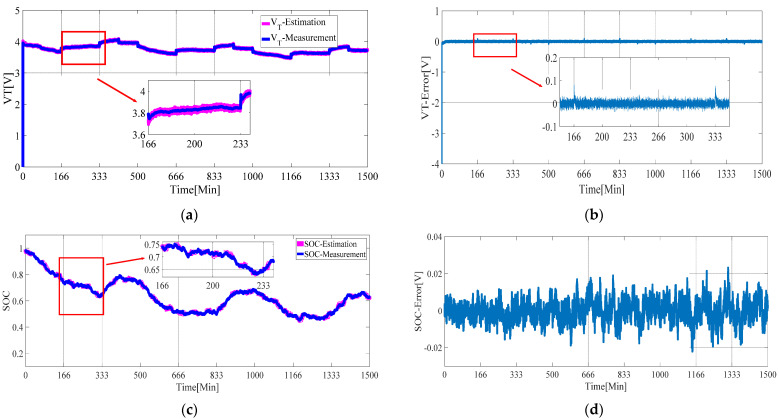
AFFRLS—EKF algorithm. (**a**) Estimated terminal voltage and measured terminal voltage; (**b**) estimated terminal voltage error; (**c**) estimated SOC and measured SOC; (**d**) estimated terminal voltage error.

Set the initial value $\theta_{0}=[0.4\;0.26\;0.22\;-0.065\;-2.0481],\;P_{0}=10^{3}*E_{5}$, where $E_{5}$ is the fifth-order identity matrix. The simulation was carried out in Matlab, and the results were shown in Figure 6 and Figure 7. Substitute the estimated results of parameters *R_S_, R_1_, R_2_, C_1_,* and *C_2_* into Equation (4) to calculate the estimated terminal voltage *V_T_*. By comparing the estimated *V_T_* with the actual measured *V_T_*, the estimation accuracy of terminal voltage can reflect the estimation accuracy of each parameter.

As can be seen from Figure 6a, the error of terminal voltage is large at several *t_i_* moments when the battery state changes, indicating that when the battery state changes, the estimation of parameters by the general least square algorithm fluctuates greatly, with the maximum error reaching 9.3789 V. When the battery state remains unchanged, the performance of the least square algorithm is high, and the estimation error of terminal voltage is kept within 0.2 V, as shown in Figure 6b. Based on parameter estimation, EKF is used to carry out SOC estimation. The SOC estimation results are shown in Figure 6c,d. At several special *t_i_* moments when the battery state changes, the parameter estimation error is large and the SOC estimation error suddenly increases. In the time interval when the battery state remains unchanged, the parameter estimation effect is good, but the SOC estimation error is relatively weak.

The estimation results of the AFFRLS-EKF algorithm proposed in this paper are shown in Figure 7.

As can be seen from Figure 7a, the estimation result of terminal voltage is very good. Even at the moment of battery state suddenly change, the estimation error of terminal voltage is less than 0.1 V, which greatly reduces the transient error of terminal voltage estimation at the moment of battery state sudden change. As can be seen from Figure 7b, the voltage estimation error is less than 0.0439 V in the battery state retention time interval. It is shown that the proposed AFFRLS-EKF algorithm can not only improve the dynamic performance but also improve the estimation accuracy in a steady state. It can be seen from Figure 7c that the estimation effect of SOC is very good, and the estimation error is kept within 2.3%.

## 5. Conclusions

Aiming at the inaccurate estimation of battery parameters caused by the change of current charging and discharging state, this paper proposes a forgetting factor adaptive least-square parameter identification algorithm. Based on the accurate identification of the second-order equivalent circuit model, the battery SOC is estimated by combining with EKF. Compared with the RLS-EKF algorithm, the algorithm proposed in this paper can better suppress the estimation error of terminal voltage when the battery state changes and improve the estimation accuracy of SOC during the steady-state.

The current flowing through series lithium-ion batteries must be strictly equal, but differences between series batteries can cause the battery to overcharge or discharge during the charge and discharge process [41,42]. This will make a significant impact on battery life, battery safety, and battery capacity utilization efficiency. In order to ensure the consistency of the voltage between the series battery cells, it is very important to balance the charge and discharge. In a BMS, a battery equalizer is used to achieve voltage consistency between series-connected battery cells. Therefore, the influence of voltage consistency between battery cells in series on the state estimation of the battery management system will be considered in the subsequent research.

## Figures and Tables

**Table 1:** Charging and discharging test data sheet.

SOC	VOC	SOC	VOC	SOC	VOC
0%	2.6936	35%	3.6504	70%	3.8270
5%	3.2567	40%	3.6776	75%	3.8627
10%	3.4552	45%	3.6982	80%	3.9073
15%	3.5220	50%	3.7184	85%	3.9628
20%	3.5408	55%	3.7414	90%	4.0056
25%	3.5750	60%	3.7670	95%	4.0510
30%	3.6144	65%	3.7954	100%	4.1000

**Table 2:** Summary of the extended Kalman filter (EKF).

1 Parameter initialization: $x_{0}=E(x_{0})$, $P_{0}=E[(x_{0}-{\hat{x}}_{0}){\left(x_{0}-{\hat{x}}_{0}\right)}^{T}]$
2 State Prediction: ${\hat{x}}_{k+1|k}^{-}=f({\hat{x}}_{k|k},u_{k})+\omega_{k}$ $P_{k+1|k}=A_{k}P_{k|k}A_{k}^{T}+Q_{k}$
3 Kalman filter gain: $K_{k+1}=P_{k+1|k}C_{k}^{T}{\left[C_{k}P_{k+1|k}C_{k}^{T}+{\hat{R}}_{k}\right]}^{-1}$
4 Measure values updated: ${\hat{z}}_{k+1}=h({\hat{x}}_{k|k})+\upsilon_{k}$
5 Posteriori estimates: ${\hat{x}}_{k+1}={\hat{x}}_{k+1|k}^{-}+K(z_{k+1}-{\hat{z}}_{k+1})$ $P_{k+1|k+1}=\left[I-K_{k+1}C_{k}\right]P_{k+1|k}$

Where $x_{0}$ represents the initial state value, $P_{0}$ represents the initial covariance, ${\hat{x}}_{k+1|k}^{-}$ represents the *k* + 1 prior estimate, $P_{k+1|k}$ represents the *k* + 1 prior covariance, $P_{k|k}$ represents the *k* prior covariance, $K_{k+1}$ represents the Kalman gain of *k* + 1, ${\hat{x}}_{k+1}$ represents the *k* + 1 posterior estimate, $P_{k+1|k+1}$ represents the *k* + 1 covariance.

## Data Availability

Data available in a publicly accessible repository that does not issue DOIs. Publicly available datasets were analyzed in this study. This data can be found here: (https://github.com/jdorsey22/298-Estimation-Theory/blob/master/EKF/DataFiles/IV_data_nonlinear.mat (accessed on 1 January 2021)).
